# Hyperbaric oxygen treatment increases intestinal stem cell proliferation through the mTORC1/S6K1 signaling pathway in *Mus musculus*

**DOI:** 10.1186/s40659-023-00444-3

**Published:** 2023-07-13

**Authors:** Ignacio Casanova-Maldonado, David Arancibia, Pablo Lois, Isaac Peña-Villalobos, Verónica Palma

**Affiliations:** 1 Laboratory of Stem Cells and Developmental Biology, Faculty of Sciences, Universidad de Chile, Las Encinas 3370, Milenio Building Floor 3, 7800024 Santiago de Chile, Nunoa Chile; 2 Present Address: Education Department, Faculty of Humanities, Universidad Mayor, Santiago de Chile, Providencia Chile

**Keywords:** Hyperbaric oxygen treatment, Intestinal stem cells, Progenitor cells, Caloric restriction, mTORC1, S6K1, Proliferation, Clarity, Rapamycin

## Abstract

**Background:**

Hyperbaric oxygen treatment (HBOT) has been reported to modulate the proliferation of neural and mesenchymal stem cell populations, but the molecular mechanisms underlying these effects are not completely understood. In this study, we aimed to assess HBOT somatic stem cell modulation by evaluating the role of the mTOR complex 1 (mTORC1), a key regulator of cell metabolism whose activity is modified depending on oxygen levels, as a potential mediator of HBOT in murine intestinal stem cells (ISCs).

**Results:**

We discovered that acute HBOT synchronously increases the proliferation of ISCs without affecting the animal’s oxidative metabolism through activation of the mTORC1/S6K1 axis. mTORC1 inhibition by rapamycin administration for 20 days also increases ISCs proliferation, generating a paradoxical response in mice intestines, and has been proposed to mimic a partial starvation state. Interestingly, the combination of HBOT and rapamycin does not have a synergic effect, possibly due to their differential impact on the mTORC1/S6K1 axis.

**Conclusions:**

HBOT can induce an increase in ISCs proliferation along with other cell populations within the crypt through mTORC1/S6K1 modulation without altering the oxidative metabolism of the animal’s small intestine. These results shed light on the molecular mechanisms underlying HBOT therapeutic action, laying the groundwork for future studies.

**Supplementary Information:**

The online version contains supplementary material available at 10.1186/s40659-023-00444-3.

## Background

Hyperbaric oxygen treatment (HBOT) consists of the administration of pure oxygen at high pressures. HBOT works under Henry’s law which states that the amount of gas dissolved in a liquid is proportional to the partial pressure that the gas exerts over such liquid. As a result, the amount of oxygen dissolved in the blood increases, which causes a systemic effect by enriching tissues in oxygen through the bloodstream.

HBOT is widely used as a treatment for diabetic foot ulcers [13, 58], brain injuries [9, 11] and to promote wound healing [24, 36]. Recent studies have proposed that its effects on wound healing occur due to the modulation of HIF-1α [60, 71] and Vascular Endothelial Growth Factor (VEGF) [40, 61] signaling pathways. Nonetheless, the HBOT mechanisms are under continuous debate and are still far from fully understood.

In the last decade, studies have focused on stem cells as cellular targets of HBOT, revealing positive effects on proliferation and regeneration on both neural [41, 70] and mesenchymal stem cell populations [23, 29]. An interesting niche for studying the effects of HBOT on somatic stem cell proliferation is the small intestinal epithelium, the most vigorously self-renewing tissue of adult mammals [3, 59]. Within this epithelium, intestinal stem cells (ISCs) reside at the base of the crypts of Lieberkühn, allowing the generation of absorptive or secretory progenitors who migrate toward the tip of the villus [4], regenerating the small intestine every three days in mice [15, 63]. The crypts of Lieberkühn have been well studied and characterized in terms of their cellular composition. ISCs can be identified by the expression of specific markers like the leucine-rich repeat-containing G protein-coupled receptor 5 (Lgr5) or Olfactomedin 4 (Olfm4). ISCs give rise to another functionally distinct population of slowly cycling stem cells called + 4 due to their position in the crypt or emergency stem cells for their capacity to respond after bacterial infections that do not possess the Lgr + mark. The complex nature of ISCs is becoming clear in recent years, revealing a hierarchy between Lgr5 + and + 4 under homeostasis and stress responses [5, 19].

The homeostatic renewal in the intestinal epithelium is commanded mainly by the oxidative metabolism and is guided by the mTOR complex 1 (mTORC1), a macromolecular nutrient- and growth factor-responsive kinase [52, 69]. This complex also integrates responses to transcription factors and oxygen [53, 55]. mTORC1 signaling involves the phosphorylation of many downstream proteins such as 4E-BP1, S6K1, and SKAR, regulating cellular anabolic growth and proliferation [10, 28]. But mTOR cante with another set of proteins forming the mTOR complex 2 (mTORC2), having potentially negative effects on cell proliferation and cell survival [54]. Therefore, mTOR signaling is central in the balance between cell proliferation, survival, anabolism, and catabolism.

A plethora of studies have shown that mTORC1 can be inhibited by the administration of rapamycin, an allosteric inhibitor 1, 8, 64] Rapamycin prevents the formation of the complex and therefore downregulates the signaling pathway [27]. Unlike mTORC1, mTORC2 is insensitive to acute drug treatment, however, when used for a prolonged time- regimen, rapamycin can also inhibit mTORC2 [57]. The effects of rapamycin over the mTOR pathway have been deeply studied by its application on cancer therapy, transplant success, and pharmacology. Yet, more recently, another interesting effect of rapamycin has been described. Rapamycin mimics a caloric restriction-like (CR) situation, resulting in an increase in life span in different species like yeast, mice, and humans due to the conservation of the pool of stem cells [16, 17, 65].

The regulatory mechanisms controlling ISC response to HBOT are just beginning to be explored. Here we analyzed the effects of acute HBOT on ISC proliferation rates of *Mus musculus* small intestine and its possible modulation by mTORC1. Our results show that animals treated for 10 days with HBOT presented a trend to increase ISCs proliferation while animals treated for 20 consecutive days exhibited a synchronized increase of proliferation among crypts and an upregulation of the mTORC1 pathway. It is worth noting that the number of cells proliferating within the crypt also increased in animals treated with rapamycin. However, the combined treatment with HBOT and rapamycin did not promote further ISC proliferation, possibly due to opposite mechanistic effects on the mTORC1/S6K1 axis. Intriguingly, our results show that HBOT can restore the inhibition of rapamycin on mTORC1. In summary, our findings suggest that HBOT can mimic the effects of rapamycin administration in a 20-day regimen, leading to similar outcomes as the CR treatment by promoting ISC proliferation but without causing the metabolic side effects associated with the drug. Therefore, we propose that HBOT may serve as a potential adjuvant treatment for intestinal injuries and pathologies.

## Results

### HBOT increases ISCs proliferation in a synchronous fashion.

**Fig. 1:**
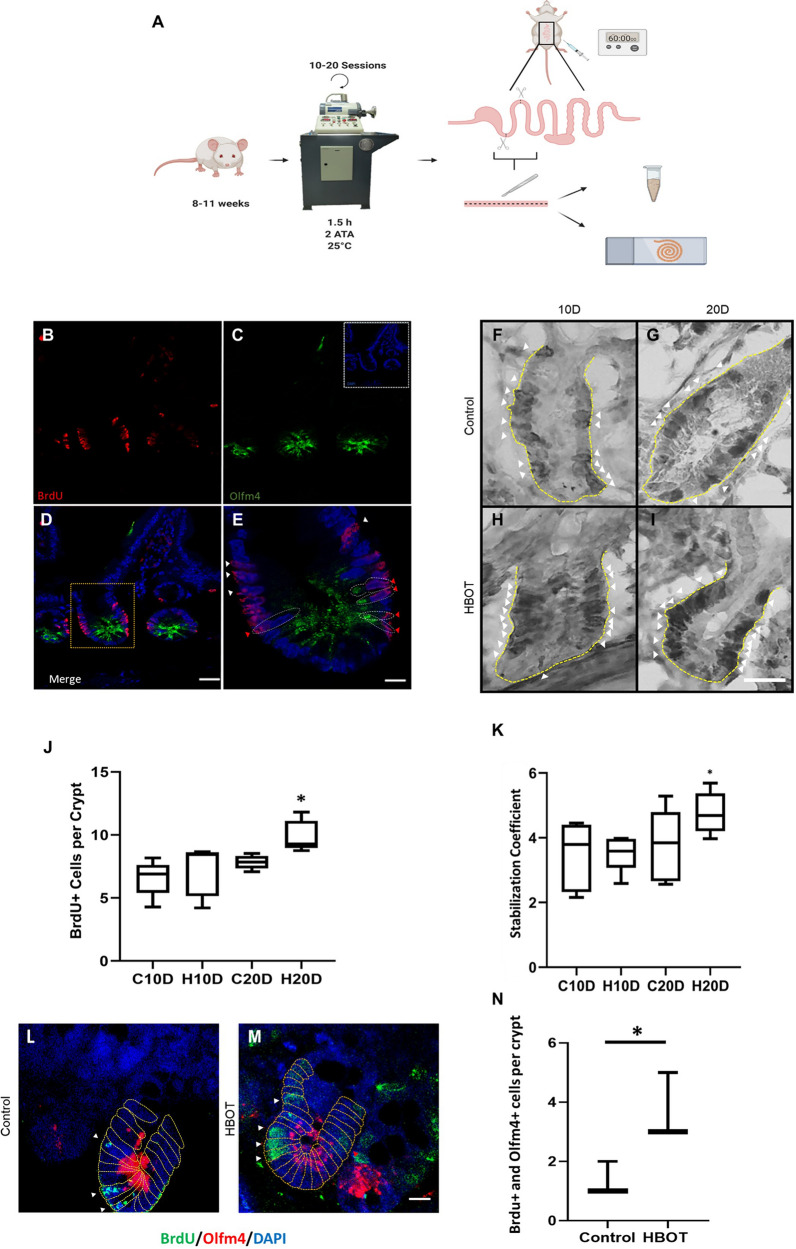
HBOT synchronously increases ISCs proliferation. **A** Schematic workflow of HBOT treatment and experimental procedures on murine small intestine samples. B-E) Representative immunofluorescence images of ISC proliferation in intestinal crypts. **B** Proliferative cells were detected by BrdU labeling (Red). **C** ISCs labeled as Olfm4 + cells, a cytoplasmatic marker (Green). The inset shows DAPI nuclear staining (Blue). **D** Merged image. The yellow box indicates a crypt with proliferating ISCs among other cell populations. Bar = 15 µm. **E** Zoom of the yellow box from image **D** Red arrowheads indicate proliferating ISCs while white arrowheads indicate other proliferating cells within the intestinal crypt. Bar = 30 µm. **F**–**G** Representative immunohistochemistry images of intestinal crypts with proliferating BrdU + cells; experimental groups as indicated. Nuclei labeled by hematoxylin counterstaining. Bar = 20 µm. Yellow dotted lines outline the crypts while white arrowheads show BrdU + cells. **J** Quantification of BrdU + cells per crypt for each treatment. Asterisks indicate statistical significance. Permutation test. < 0.05. n = 5. **K** Stabilization coefficient indicating the variation level within each group. Only H20D shows significant differences, indicating synchronicity. Permutation test. < 0.05. n = 4. **L**, **M** Representative immunofluorescence images of control and HBOT intestinal crypts. The dashed line shows cellular limits. White arrowheads show cells positive for both BrdU and Olfm4 markers. Bar = 25 µm. **N** Quantification of BrdU + and Olfm4 + cells within the crypt between control and HBOT groups (for 20 days). n = 3. Asterisks indicate statistical significance. < 0.05

Stem cells in vivo reside in a dynamic and specialized microenvironment, the so-called niche. The stem cell niche is influenced by a variety of factors including physical and metabolic parameters. Oxygen tension is known to be an important cellular input modulating stem cell self-renewal and differentiation potential. Thus, many stem cells, including ISCs, respond actively to changes in oxygen tension. To evaluate the impact of oxygen on ISCs we used young mice (8–11 weeks) treated with 10 or 20 sessions of 1.5 h each at 2 ATA and 25 °C. After the last HBOT session, the animals were injected intraperitoneally with BrdU for one hour to label only the proliferative cell population within the crypt [56]. The duodenum was subsequently collected and longitudinally divided to obtain samples for Western blot and immunohistochemistry analyses (Fig. 1A).

ISCs are located at the bottom of the intestinal crypt and are positive for the proliferative marker BrdU (Fig. 1B–E). By evaluating the number of BrdU + cells per crypt in all experimental groups, we revealed that mice treated with HBOT for 20 days (H20D group) showed an increment in proliferating cells (Fig. 1F–J). Moreover, HBOT increased the proliferation within the crypts in a synchronized fashion in the H20D group. Calculating the stabilization coefficient (Fig. 1K) for each group confirmed that this group was the only one showing significant differences in this parameter. By examining the co-distribution of BrdU + and the ISC cell marker Olfm4 + , we confirmed that HBOT increases ISC proliferation (Fig. 1L–N).

Interestingly, other cells, close to the intestinal crypts, also exhibited BrdU staining. Noteworthy, HBOT also increases the total number of cells within the crypt (Additional file 1: Fig. S1). Hence, we cannot discard that HBOT affects another ISC population, besides the Olfm4 + pool.

### HBOT has an accumulative effect in time.

**Fig. 2:**
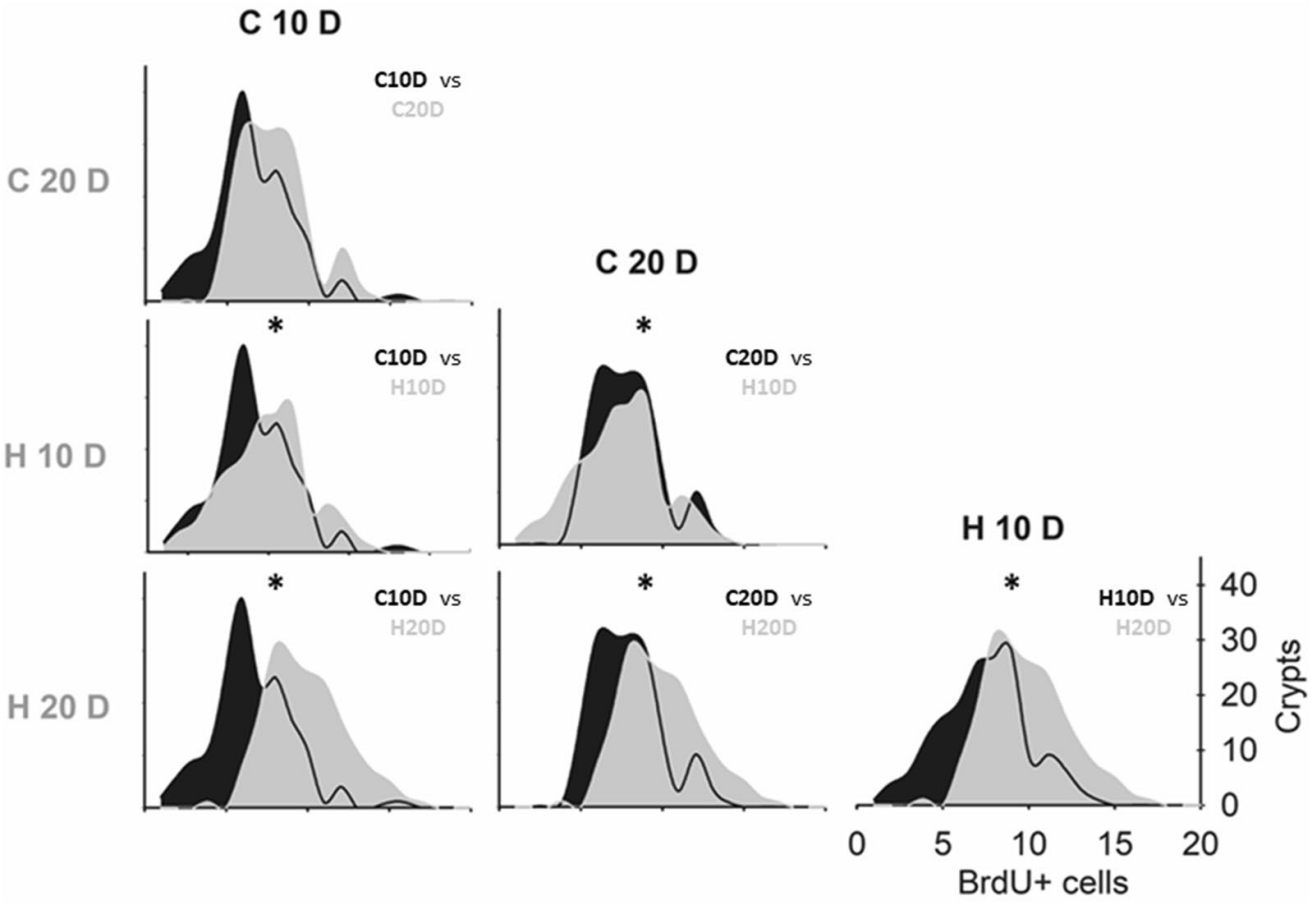
HBOT has an accumulative effect in time. Comparison of the frequency distribution of BrdU + cells per crypt between groups as indicated. Asterisks indicate significant differences (< 0,05). Kolmogorov–Smirnov test. n = 4

The frequency distribution from the group treated with HBOT for ten days (H10D group) differed from its control group (C10D group) and H20D despite not showing significant differences in the number of BrdU + cells per crypt. When we analyzed the frequency distribution per group (Fig. 2), we found that only control groups showed no differences between them (p = 0.796). The latter indicates that, despite not showing significant differences in the number of BrdU + cells per crypt, HBOT already generates a change in the proliferation behavior of the cells within the crypts at ten days of treatment.

### Acute HBOT does not change the small intestine’s enzymatic activity.

**Fig. 3:**
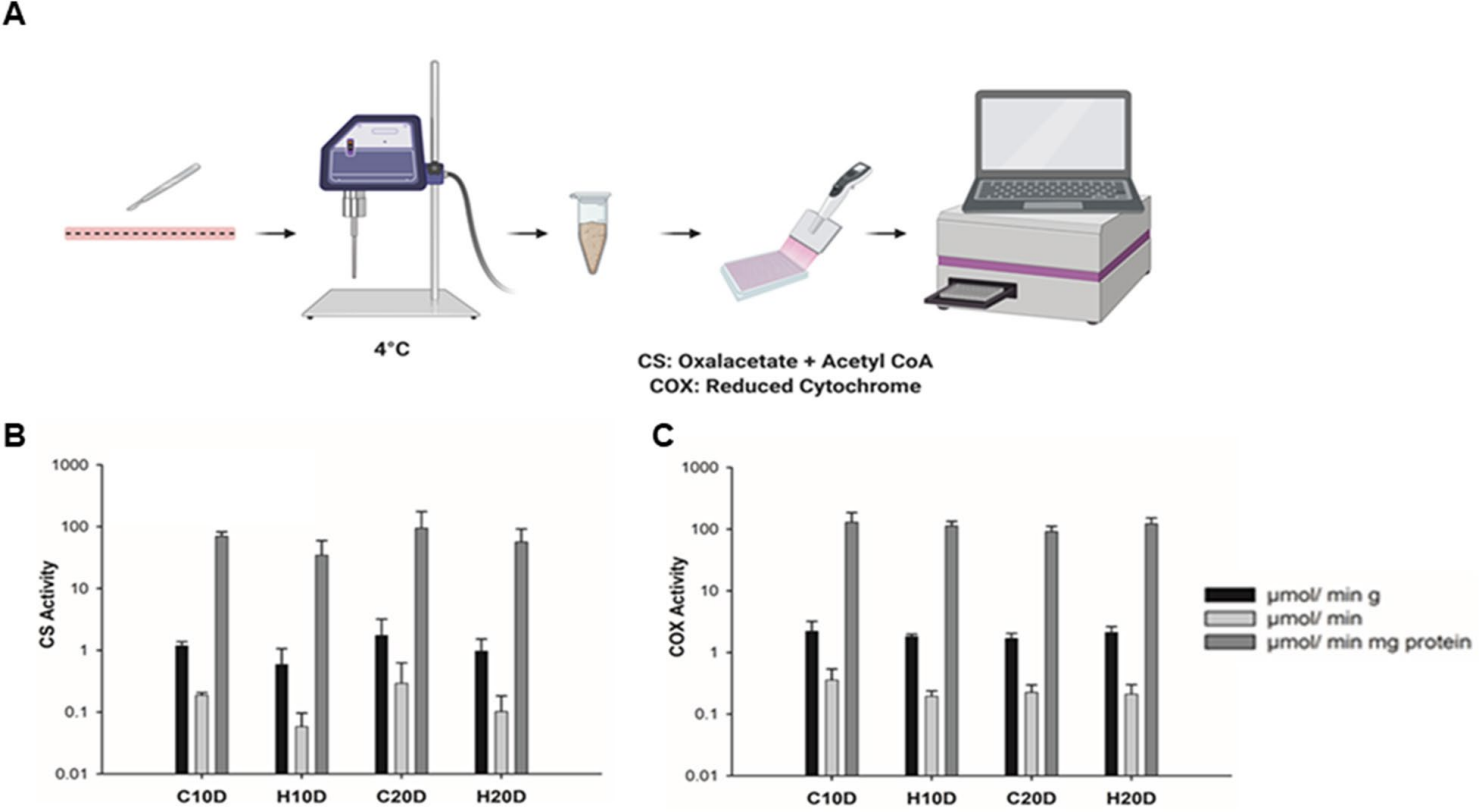
Acute HBOT does not affect enzymatic activity in the small intestine. **A** Workflow of the enzymatic assay for CS and COX activity in small intestine tissue. **B** Bar graph of CS activity (μmol/min) under each treatment. No significant differences were found. (> 0.05). n = 3. **C** COX activity (μmol/min) under each treatment. No significant differences were found. Black bars indicate activity per gram of tissue, dark grey bars indicate activity per total proteins in the sample and light grey bars indicate total activity. (> 0.05). n = 3

We next sought to analyze if the increment in the proliferation of the ISCs within the crypts by HBOT could be the result of an increase in the activity of cytochrome c oxidase (COX) and citrate synthase (CS), both enzymes related to aerobic ATP generation. To this end, we measured the activity of these enzymes in the whole duodenum in each experimental group (Fig. 3A). No differences were found the enzymatic activity of COX and CS when analyzed per gram, total tissue, or total protein (standardized by Bradford assay) between the control (C10D and C20D group) and both HBOT groups (Fig. 3B, C). Therefore, HBOT does not affect enzymatic activity related to oxidative metabolism in the small intestine, implying that it must encompass another molecular pathway to account for its effect.

### HBOT modulates ISCs proliferation through mTORC1 pathway activation.

**Fig. 4:**
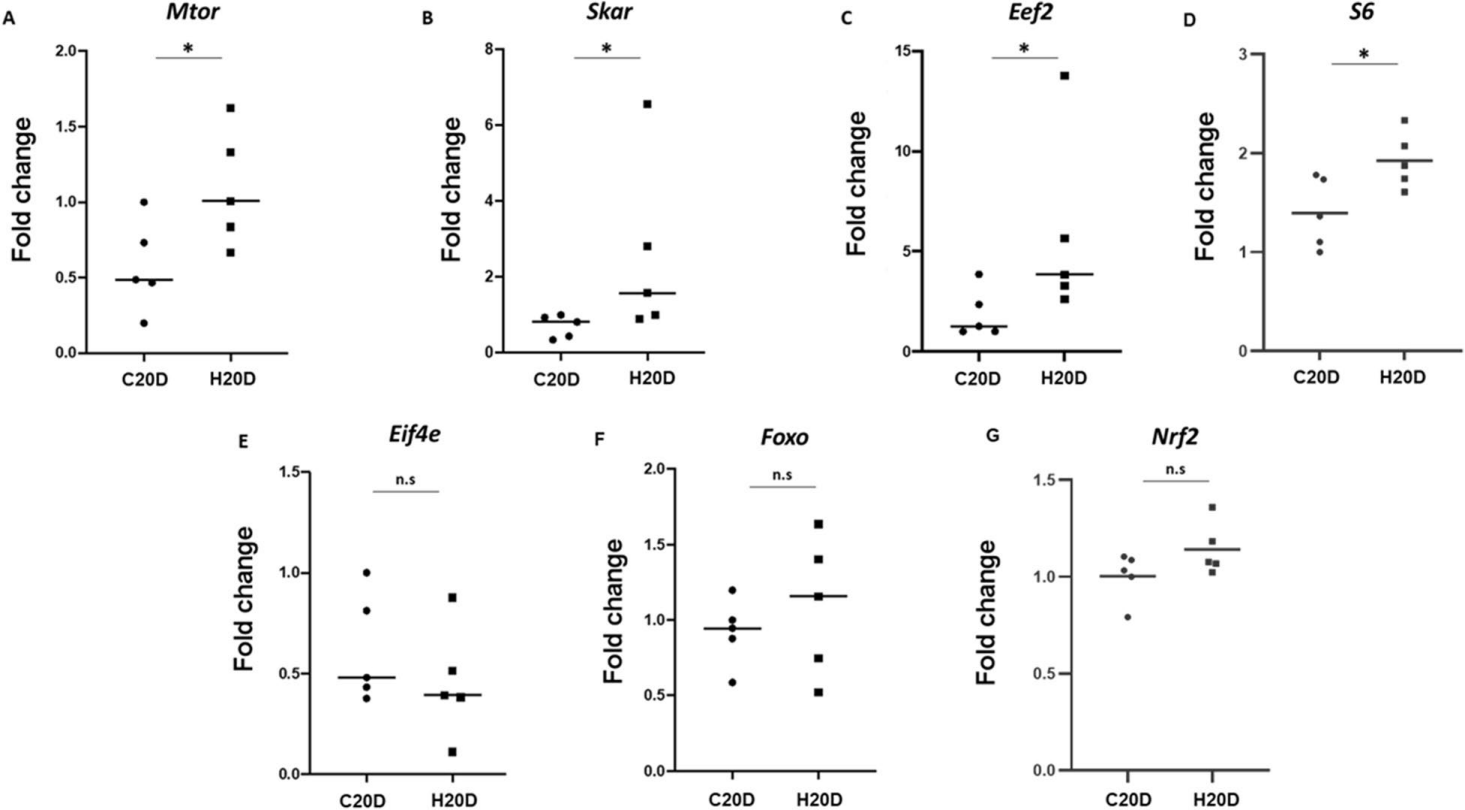
HBOT enhances the mTORC1/S6K1 axis. **A** *Mtor* mRNA expression in C20D and H20D conditions (p = 0.02). **B** *Skar* mRNA expression in C20D and H20D conditions (p = 0.02). **C** *Eef2* mRNA expression in C20D and H20D conditions (p = 0.01). **D** *rpS6* mRNA expression in C20D and H20D condition (< 0.05). **E** *Ei4fe* mRNA expression in C20D and H20D conditions (> 0.05). **F** *Foxo1* mRNA expression in C20D and H20D conditions (> 0.05). n = 5. **G** *Nrf2* mRNA expression in C20D and H20D condition (> 0.05). The expression graphs of all genes were normalized using the housekeeping gene *GAPDH*, with the exception of *Nrf2* and *rpS6*, which were normalized using the mean expression of two housekeeping genes (*GAPDH* and *B2M*)

A growing body of literature describes mTORC1 signaling as the prototypical pathway regulating cell proliferation in ISCs. To evaluate if the effects of HBOT for 20 consecutive days were mediated by the mTORC1 pathway, we evaluated the expression levels of different genes related to mTORC1 signaling through qPCR analysis. Our results show that HBOT increased the expression of several genes related to the activation of the mTORC1/S6K1 axis such as *Mtor*, *Skar,* and *Eef2* (Fig. 4A–C), as well as the ribosomal *S6*, (Fig. 4D) but did not affect the mTORC1/4E-BP1 axis as the expression of *Ei4fe* was not altered (Fig. 4E). On the other hand, HBOT did not affect the mTORC2/SGK1 axis, as the expression of *Foxo1* in H20D shows no variation in comparison to C20D (Fig. 4F). Nrf2, which has been reported to regulate mTOR expression and also has been associated with HBOT [22], showed a slight increase after HBOT although the difference was not significant when compared to the control condition (Fig. 4G).

### HBOT reverses the inhibitory action of rapamycin on mTORC1.

**Fig. 5:**
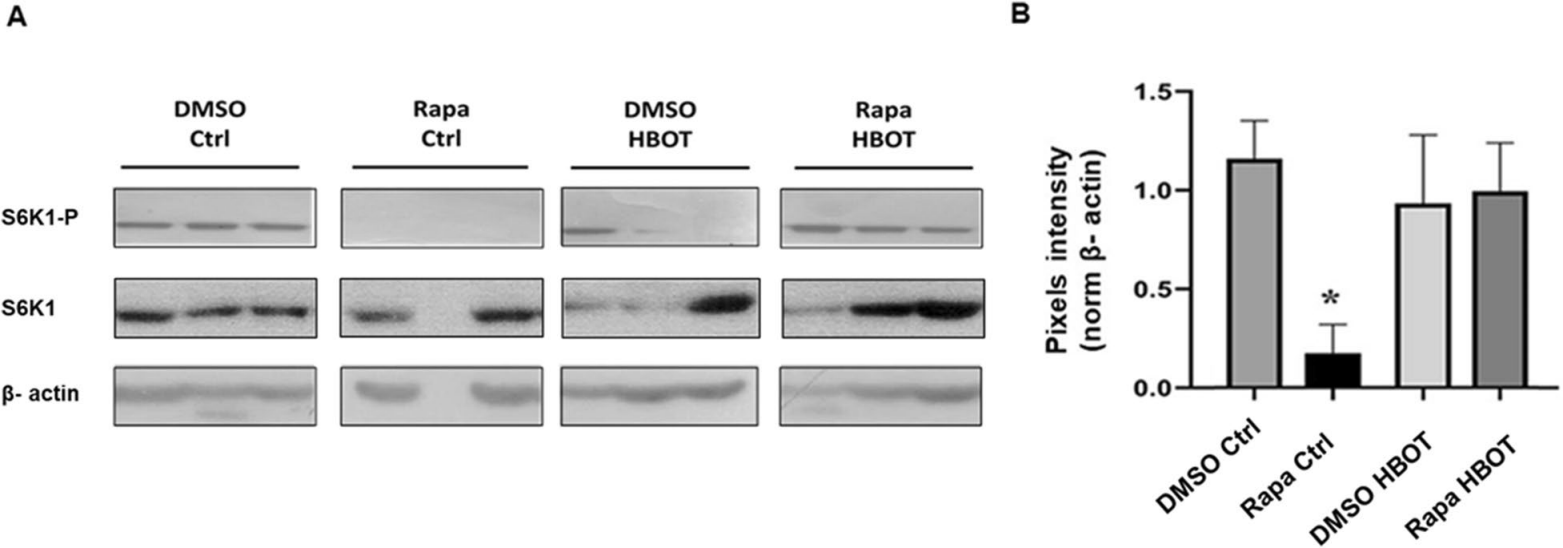
HBOT recovers rapamycin-induced mTOR pathway inhibition. **A** Western blot for phosphorylated S6K1 (S6K1-P) and β-actine in each experimental group (triplicate). The first row shows DMSO control group. The second row shows the rapamycin control group. The third row shows the rapamycin and HBOT groups. The last row shows the DMSO and HBOT groups. HBOT sessions for 20 days. **B** Quantification of pixel intensity for S6K1-P presence in each group normalized to internal control β-actine. Asterisks indicate statistical significance. Permutation test. < 0.05, n = 4 per group

In order to validate the activation of mTORC1 by HBOT, we conducted four additional experimental groups by combining 20 sessions of HBOT with or without rapamycin treatment (Table 2). Protein synthesis, which is regulated by mTORC1 through the phosphorylation of S6K, is essential for translation initiation. Consistent with previous studies, animals treated with rapamycin exhibited suppression of the mTORC1 pathway, as evidenced by the lack of phosphorylation on S6K1 (Fig. 5A and Additional file 2: Fig. S2). Additionally, animals treated with rapamycin vehicle (DMSO) showed no significant change in the phosphorylation status of S6K1, either with or without HBOT. Remarkably, animals that received combined treatment with rapamycin and HBOT (Rapa HBOT group) showed a consistent phosphorylation on S6K1 (Fig. 5B) with no significant difference to DMSO Ctrl or DMSO HBOT, indicating that HBOT can counteract rapamycin-induced inhibition of mTORC1 pathway signaling.

**Fig. 6:**
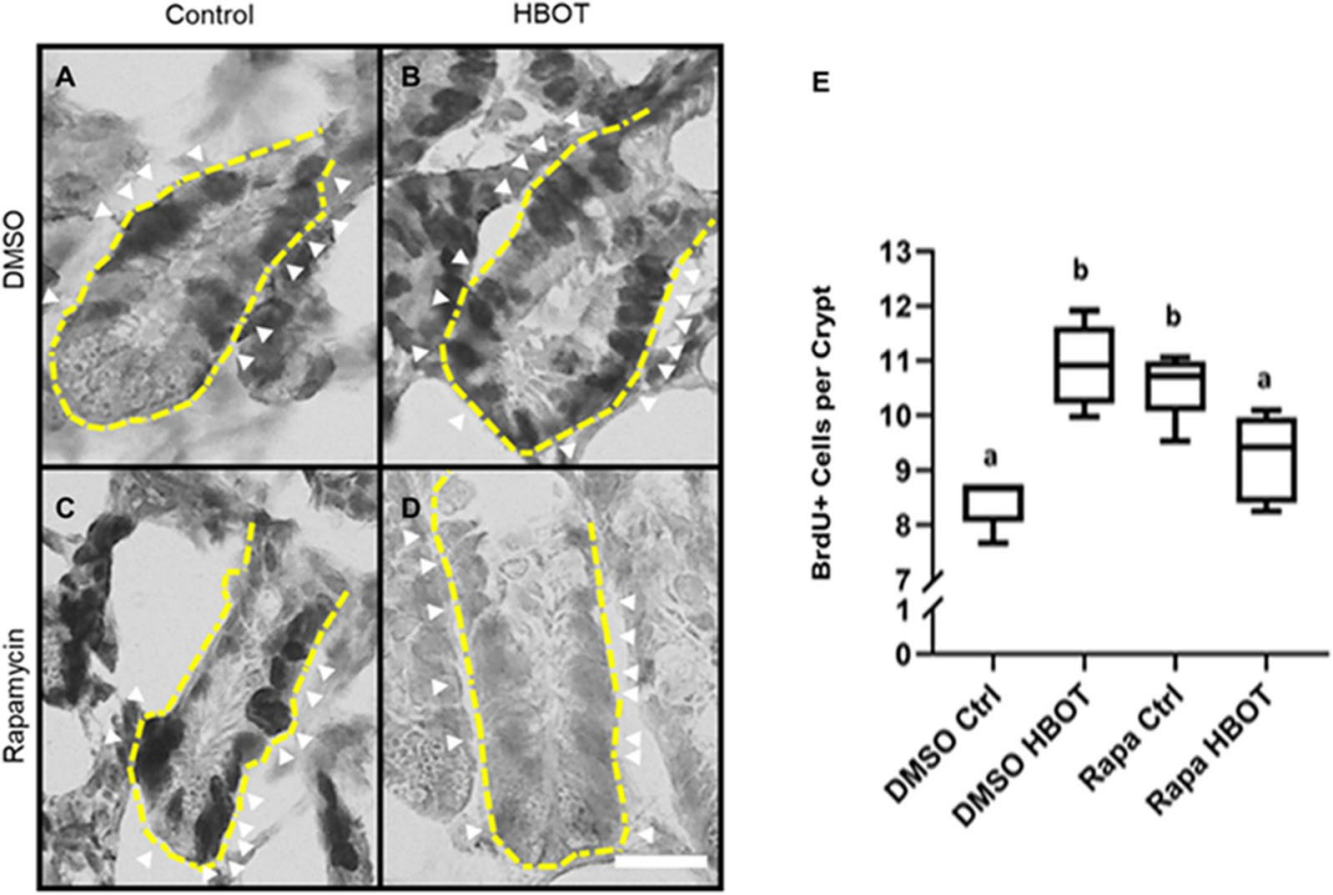
HBOT and rapamycin increase ISCs proliferation independently.** A**–**D** Representative immunohistochemistry images for proliferating cells, labeled by BrdU incorporation, in intestinal crypts for each experimental group. 20 days of treatment in each group. Nuclei labeled by hematoxylin counterstaining. Bar = 20 µm. Yellow dotted lines outline the crypt periphery. White arrowheads show BrdU + cells. **E** Quantification of BrdU + cells for each treatment. The same letters indicate no statistical significance while different letters indicate statistical significance. Permutation test. < 0.05. n = 5 per group

Previous studies have shown that mTORC1-mediated phosphorylation of S6K1 is enhanced by CR, promoting ISC proliferation [32, 33]. The effects of acute HBOT on ISC proliferation observed in our study suggest that it can simulate the effects of CR. Rapamycin is considered a potential CR mimetic. Indeed, the comparison of the effects of rapamycin treatment (Rapa Ctrl group) and 20-day HBOT treatment (DMSO HBOT group) on ISC proliferation indicated that both treatments increased the number of BrdU + cells per crypt (Fig. 6A–E) in the same way. However, the combination of HBOT and rapamycin (Rapa HBOT group) did not result in an additional increase in ISC proliferation, being no different to the control group (DMSO Ctrl).

**Fig. 7:**
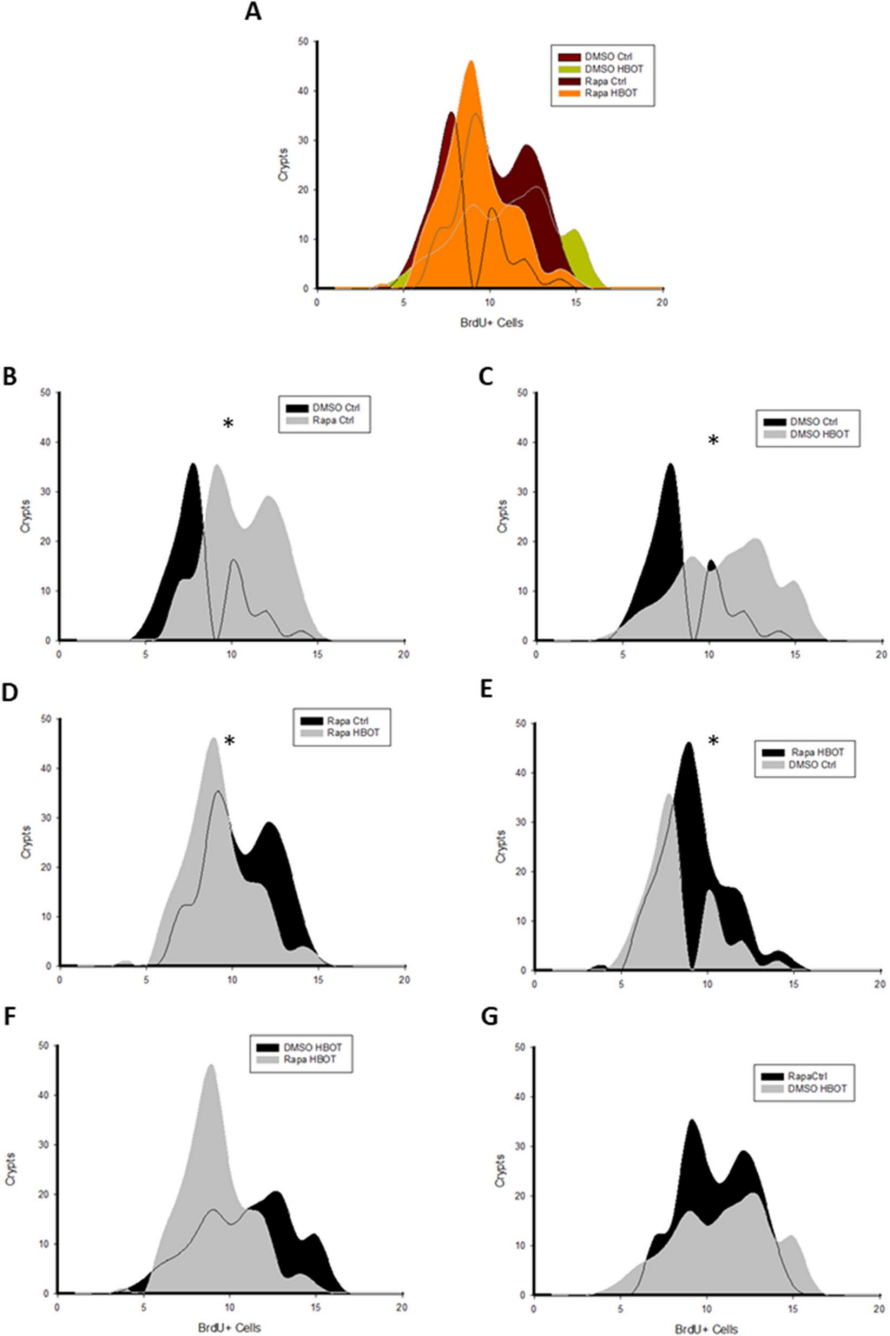
HBOT can simulate a caloric restriction. **A** Superposition of frequency distribution for each group. All comparison was made by a Kolmogorov–Smirnov test. **B** Comparison between DMSO Ctrl and Rapa Ctrl. p = 0.004 **C** Comparison between DMSO Ctrl and DMSO HBOT. p = 0.003 **D** Comparison between Rapa Ctrl and Rapa HBOT. p = 0.013 **E** Comparison between DMSO Ctrl and Rapa HBOT. p = 0.009. **F** Comparison between Rapa HBOT and DMSO Ctrl. > 0.05. **G** Comparison between Rapa Ctrl and DMSO HBOT. > 0.05. n = 5

Finally, we conducted a frequency distribution analysis of BrdU + cells per crypt to compare among treatments. Our findings indicated no significant differences between the Rapa HBOT and DMSO Ctrl groups (Fig. 7F) and between the Rapa Ctrl and DMSO HBOT groups (Fig. 7G). These observations, plus the results from Fig. 6E, suggest that HBOT has a similar effect on ISC proliferation as rapamycin under the given experimental conditions.

**Fig. 8:**
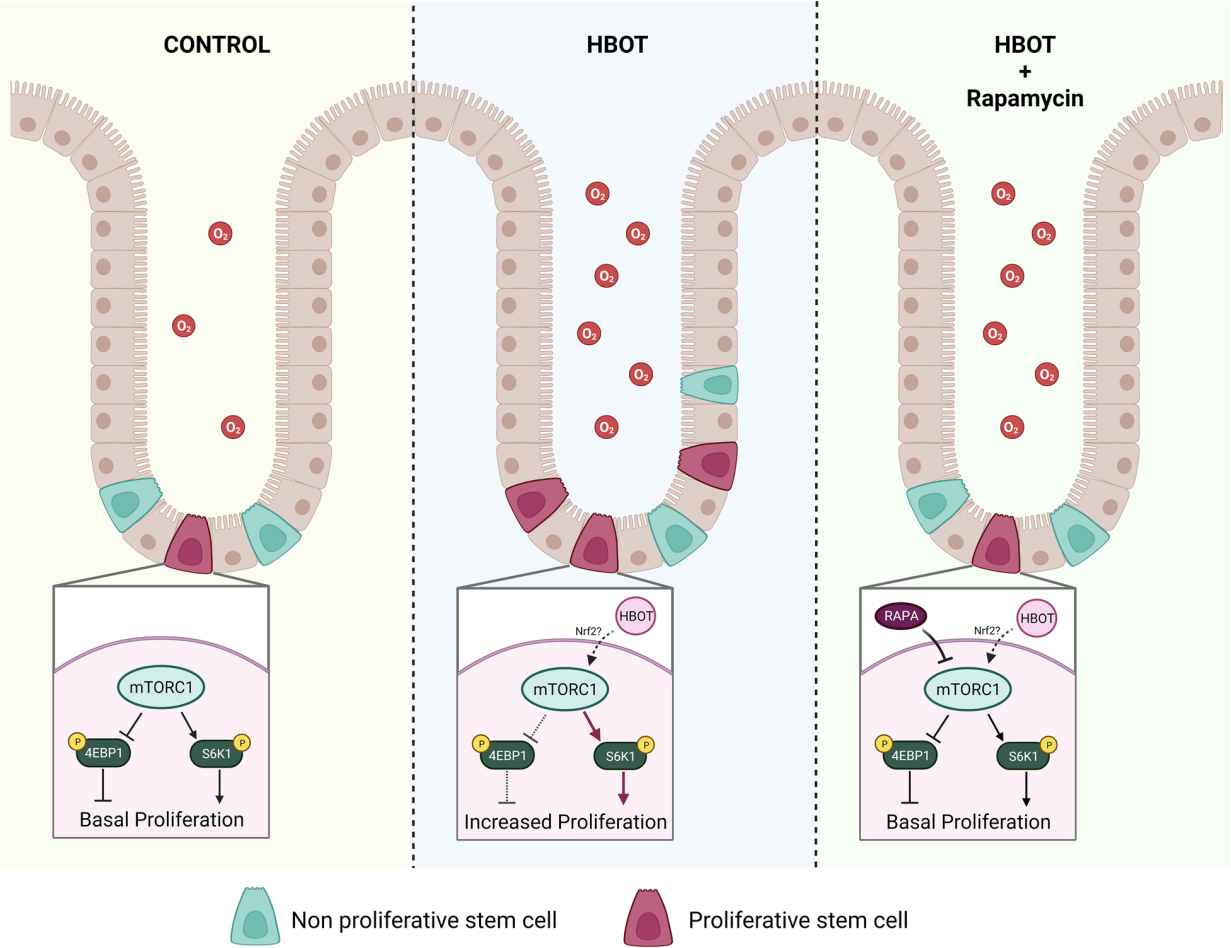
Graphical representation of mTOR regulation by HBOT. Left panel: Control condition, characterized by normal oxygen concentration and basal proliferation of ISCs. Middle panel: HBOT condition, featuring increased oxygen concentration and enhanced mTORC1 activity, potentially mediated by Nrf2. Right panel: Combined HBOT and Rapamycin administration, exhibiting higher oxygen concentration (compared to control) but lacking enhanced mTORC1 activity due to opposing effects of rapamycin and HBOT on mTOR signaling. Brown cells depict epithelial cells, green cells represent non-proliferative intestinal stem cells, red cells represent proliferative intestinal stem cells, and red circles symbolize oxygen molecules

Our findings strongly indicate that 20 days of HBOT administration enhances the proliferation of ISCs mediated by mTORC1, acting through the S6K1 axis. Moreover, HBOT reverses the inhibitory action of rapamycin on mTORC1, demonstrating an opposite outcome on mTORC1 activation (Fig. 8).

## Discussion

Despite the beneficial metabolic effects described for HBOT in several medical conditions, the underlying molecular causes are not entirely clear. HBOT has been reported to modulate somatic stem cells’ proliferative behavior in mesenchymal stem cells in the bone marrow [29] and neural stem cells within the brain [66, 68]. In this study, we show that HBOT can synchronously enhance the proliferation of ISCs and provide findings that the mTORC1/S6K1 axis mediates this proliferative effect.

Nearly 90% of the intestinal epithelium is replaced every 3–4 days by newly generated cells from the crypt epithelium. However, long-lived ISCs harbor in the crypt bottom to replenish a large amount of the disposable functional epithelium. ISCs produce rapidly cycling progenitor cells that migrate up the crypt-villus axis and differentiate into mature epithelial cells that are eventually shed off into the lumen. The small intestine partial pressure of oxygen values fluctuates between 2–5%, 3–6%, and 5–9% O_2_ for the lumen, mucosa, and serosa layers, respectively [25], but under HBOT conditions, these levels can rise to 22% in tissue with direct contact with air [14], increasing oxygen availability in the tissue.

### HBOT and metabolism

The increase in oxygen availability does not translate as an increase in ATP production due to an enhanced oxidative metabolism in the intestinal tissue, as revealed by the analysis of the enzymatic activity of COX and CS. These results were in concordance with previous reports of mitochondrial oxygen consumption [37, 67] and our previous work, where we showed that HBOT for ten consecutive days did not change the basal metabolic rates in mice [49]. They are also consistent with a recent in vitro analysis of lung fibroblast treated with HBOT, reporting no changes in CS activity [20]. Notwithstanding, it is remarkable how a treatment that increases oxygen tension does not alter oxidative metabolism in the intestinal epithelium, while other reports indicate that acute HBOT does generate changes in tissues like the brain or skeletal muscle [34, 45], indicating that the response to HBOT is tissue dependent.

### *HBOT increases BrdU* + */Olfm4* + *cells within the crypt*

In our previous work, we exposed mice to HBOT once daily for ten consecutive days and did not find a significant effect on ISCs proliferation. All groups had similar numbers of BrdU + cells per crypt, nonetheless, revealing a tendency to increase proliferation. In the present work, increasing the time length of treatment by applying HBOT for 20 consecutive days led to a significant cell proliferation rise within the crypts. Of note, 20 sessions are still considered an acute treatment [14].

Although the impact of HBOT is only significant on day 20, the accumulative effect is already evident on day 10, as differences in the frequency of proliferative cells were observable. The latter indicates that HBOT for ten consecutive days is sufficient to generate an incipient change in the proliferative behavior of the cells within the crypts (See Figs. 1J, K and 2).

Cells proliferating within the crypt corresponded mainly to ISCs, as evidenced using the specific ISC marker Olfm4, revealing co-distribution with the proliferation marker BrdU. Notwithstanding, another cell population showed proliferative labeling but no Olfm4 signal. Based on their position within the crypt, we pose that these cells correspond to proliferating progenitor cells at the moment of the BrdU pulse. But we cannot discard the possibility that HBOT could stimulate the proliferation of emergency ISCs (See Fig. 1E). The recruitment of these cells together with ISCs would represent an interesting broader effect of this treatment. HBOT has been recently reported to increase the proliferation of progenitor cells in vitro [7]. To our knowledge, our result would be the first to describe similar data in vivo in the intestinal niche. In addition, if one of the targets of acute HBOT is the so-called emergency ISCs, a population described as quiescent [47], we cannot rule out that the treatment can promote their recruitment to ISCs. The possible relationship between the quiescent and the actively cycling ISCs and the biological consequences of their HBOT response needs to be further explored.

The increase in cell proliferation within the crypt after HBOT for 20 consecutive days occurred synchronously. Meaning that throughout the duodenum, all the animals’ intestinal crypts in the H20D group presented a similar and higher number of BrdU + cells than the control or H10D groups. This synchronicity could emerge as a consequence of cell cycle arrest generated by HBOT or by the temporal maximization of the proliferative capacity of the crypt’s cells. As shown previously, establishing the BrdU and Phosphorylated Histone-H3 (PHH3) proportion allows for evaluating of cell cycle arrest [12, 50]. Based on those studies, we analyzed the presence of these proliferative markers in the C20D and H20D groups and found that HBOT does not arrest the cells in a particular cell cycle stage (data not shown). Thus, we suggest that HBOT maximizes the proliferation potential in the intestinal crypts in an acute fashion.

### HBOT and the mTOR pathway

Here we show that HBOT for 20 consecutive days promotes cell proliferation through the mTORC1/S6K1 axis. The ability of HBOT to activate the mTORC1/S6K1 axis also stimulates protein synthesis by increasing mTOR itself (as seen in Fig. 4A), driving the translation elongation through eEf2 [21, 62] and promoting higher efficiency in protein translation and ribosome biogenesis by SKAR [38, 43, 51]. Furthermore, the ribosomal protein S6, which is directly involved in protein synthesis [46], also exhibits an increase in mRNA expression. Although the phosphorylated conformation is primarily associated with this action, the observed increase in *S6* can still correlate with the biological effect on cell proliferation. All this without the activation of mTORC1/4E-BP1 axis or modifying the mTORC2 axis, as evidenced by *Foxo* expression levels (Fig. 4E). These results agree with the results shown by Saxton and Sabatini relating the activation of the mTORC1/S6K1 axis with an increased protein synthesis leading to cell proliferation and migration [53]. Of note, the lack of activation of the mTORC1/4E-BP1 axis by HBOT turns out to be advantageous, as many investigations related to cancer indicate that activation of the eIF4e axis can lead to tumorigenesis and tumor progression [2, 30, 31]. Similarly, the literature suggests that mTORC1 can enhance the activity of Hif-1α through the 4E-BP1-eIF4e axis [39]. However, it has also been reported that HBOT can augment *HIF-1α* levels and its activity in intestinal samples [48]. Therefore, the association between mTOR and HIF-1α and the impacts of HBOT on HIF-1α expression are intricate and influenced by multiple factors that require consideration in future investigations.

### mTORC1 and rapamycin

The inhibition of mTORC1 through rapamycin administration (Rapa Ctrl group) showed an increment in the number of BrdU + cells per crypt. This effect can be attributed to the capacity of rapamycin to induce a CR-like situation, generating changes in the ISCs niche due to paracrine signaling from Paneth cells to ISCs which in turn stimulates proliferation as a short-time response [32]. Fasting, acute gut stress, inhibits the mTORC1 pathway in diverse species. Of note, rapamycin inhibits mTORC1 principally through the S6K1 signaling axis [26].

HBOT elevates the levels of Nrf2, a protein that modulates the expression of antioxidant response elements [22]. In addition, Nrf2 can regulate the expression of mTOR [6]. This correlation could potentially explain our findings, as demonstrated by the enhanced expression of *mTOR* following HBOT, as shown in Fig. 4A. This increase in mTOR expression may effectively counteract the inhibitory effects of rapamycin on mTOR activity, as illustrated in Fig. 5A. When evaluating *Nrf2* within the intestine at day 20, we observed no significant differences but a clear trend towards an increase in the HBOT group. This observed tendency, in conjunction with the effects observed in our study, suggests that analysis at day 20 may be too late. Nevertheless, we propose that HBOT could regulate mTOR expression through Nrf2. Upregulation of mTOR by HBOT, followed by its suppression by rapamycin, may explain the lack of increased cell proliferation within the crypt when HBOT and rapamycin are applied together, as each treatment individually would yield different outcomes.

The effects of acute rapamycin treatment have shown to increase the lifespan of ISCs through an increase in autophagy, being mediated by changes at the level of chromatin organization as a result of the mTORC1/eIF3 pathway [35, 42, 44]. eIF3 regulates the phosphorylation of S6K1 that, in turn, signals downstream. We found that HBOT treatment induced a similar response in ISCs as observed with a CR-like situation mediated by rapamycin. Notably, HBOT is a non-invasive and pain-free treatment with reported beneficial effects in other contexts. Our findings suggest that HBOT treatment may influence the expression of eIF3 and promote autophagy in the intestinal niche, but further research is required to confirm this proposed mechanism.

## Conclusions

HBOT for 20 consecutive days potentiates intestinal crypt cells’ proliferative behavior. Such an increase in proliferation occurs synchronously in the duodenum without altering the oxidative metabolism of the intestinal tissue, suggesting that HBOT maximizes the proliferation rate of cells within the crypt for a short time frame. Our results show that mTORC1/S6K1 pathway modulation is responsible for this HBOT-driven increase in proliferation. When rapamycin inhibits mTORC1, ISCs undergo a CR-like situation, which in mice generates a similar effect to that observed if treated with HBOT for 20 consecutive days. However, when combined treatments do not affect ISC proliferation, most likely as a consequence of their opposite action on the mTORC1/S6K1 axis. While HBOT and rapamycin treatment may have similar effects on ISC proliferation, they may affect the mTORC1 pathway quite differently. Our results shed light on the molecular mechanisms underlying HBOT therapeutic action, a medical treatment used for a long time but whose subjacent mechanisms on stem cells remain unclear.

## Methods

### Animals

Forty-six adult (3 months old) males of the BALB/c strain of *Mus musculus* were obtained from the central animal housing facilities at the Faculty of Sciences. All animal procedures followed Chilean legislation and were approved by Institutional Animal Care and Use Committee (CICUA) at the Universidad de Chile. The study was carried out in compliance with the ARRIVE guidelines.

To assess the effects of HBOT on ISCs proliferation, we generated four groups: Control group for ten days (C10D), HBOT group for ten days (H10D), Control group for twenty days (C20D), and HBOT for twenty days (H20D) (Table 1). Each group consists of five animals, considering no siblings in the same group. Mice were housed with their siblings but properly identified and kept in a temperature-controlled room, maintained at 25° ± 1 °C in an LD = 12:12 cycle, with food (Prolab RMH 3000, LabDiet) and water ad libitum.

**Table 1:** Animal groups generated for analysis of HBOT impact on ISCs proliferation

	Days	
	10 Days	20 Days
Treatment		
Control	C10D	C20D
HBOT	H10D	H20D

To explore the relationship of HBOT with mTORC1 signaling, we generated another set of 4 experimental groups: 2 Groups without HBOT sessions but injected with rapamycin (Rapa Ctrl) or with DMSO (vehicle of rapamycin) (DMSO Ctrl), and 2 groups treated with HBOT injected with rapamycin (RAPA HBOT) or with DMSO (DMSO HBOT) (Table 2). All groups were treated for 20 consecutive days. Each group included five animals maintained in the same conditions as described above.

**Table 2:** Animal groups generated for the study of the relationship between HBOT, mTORC1 and ISCs proliferation

	Treatment	
	Control	HBOT
Pharmacology		
DMSO	DMSO Ctrl	DMSO HBOT
Rapamycin	Rapa Ctrl	Rapa HBOT

### HBOT sessions

HBOT was performed in a 19.56 L experimental chamber at 25ºC (Osorio Hermanos & Cia. Ltd., Quillota, Chile). Animals were placed in individual cages, then the remaining air inside the chamber was replaced with 100% O_2_ while the pressure was increased for 15 min until 2.0 ATA (absolute atmospheres) were reached. The latter conditions were maintained for 1.0 h followed by decompression from 2.0 ATA to atmospheric pressure gradually for another 15 min (Fig. 1A). Therefore, each session took 1.5 h [18, 49].

### Rapamycin administration

Rapamycin (Calbiochem, 553210) was dissolved in DMSO (5 mg/125 µL) and diluted in PBS 1X 1:100 v/v. Every other day rapamycin was injected intraperitoneally (2 mg/kg), always at the same hour and before starting the HBOT session.

### Intestinal tissue dissection

After the last HBOT session animals were injected intraperitoneally with 80 µl of 20 mg/mL BrdU (Sigma-Aldrich). One hour later the animals were sacrificed by cervical dislocation. Immediately, the entire digestive tract was dissected on a cooled surface and the intestine contents were gently removed mechanically. Next, the first third of the small intestine (duodenum) was cut longitudinally and used for histological analysis. This segment was fixed in 4% PFA (Sigma-Aldrich) for 2 h at 4 °C followed by dehydration overnight (O.N) in 30% sucrose (Merck) solution. Tissues were embedded in OCT (Tissue-Tek), placed in a disposable vinyl specimen mold (Tissue-Tek Cryomold), and stored at -80 °C until further use.

### Immunostaining

Immunofluorescence (IF) and immunohistochemistry (IHC) were performed as described in Peña-Villalobos et al. [49]. Briefly, slides of either 14 or 7 µm thickness were prepared with the epitope-unmasking protocol and incubated overnight at 4 °C with 1:100 monoclonal mouse Anti-Bromodeoxyuridine Clone Bu20a (BrdU, Dako, M0744) primary antibody or 1:400 monoclonal rabbit Anti-Olfm4 (Cell Signaling, 39141). For IF, goat anti-mouse Alexa 555 (1:500 Life technology, A21424) and goat anti-rabbit Alexa 488 (1:500, Life technology, A11034) were used as secondary antibodies. DAPI (Invitrogen) was used to label nuclei. For IHC, hematoxylin/eosin (H&E) and DAB staining were performed as described in Peña-Villalobos et al. [49]. To analyze the IHC, an optical microscope (Olympus BX51) at 40X and 100X equipped with a digital camera (Moticam 2500) was used whereas for IF a confocal microscope (Zeiss 710) was used. Z-stacks and image analysis were performed with ImageJ software and FIJI distribution. Figures were created with Biorender.

### Intestinal clearing

Duodenum was cut transversely in 2 mm thick sections, fixed in PFA 4% O.N. and dehydrated in methanol in PBS 1 × until methanol was 100%. Then samples were transferred to a clarity solution (4% acrylamide, 0.05% Bis-acrylamide, 0.25% VA-044 in PBS) and let polymerize for 3 h at 37 °C. After washing twice in PBS-triton 0.5% samples were transferred to a clearing solution (4% SDS, 0.1 M boric acid, pH 8.5) for five days at 37 °C in constant agitation. For immunolabelling, the samples were blocked for 2 h in 5% serum in PBS triton 0.1% and incubated with the primary antibody in 3% serum and 5% DMSO in PBS triton 0.1% for three days. Finally, samples were rinsed for 1 h three times and incubated with the secondary antibody for two days.

The samples were positioned on a glass bottom petri dish covered with PROTOS solution (refraction index 1.46) and observed in confocal microscopy (Zeiss 720) using tiles function with the 4X objective. 2D and 3D images were generated using the free license of FIJI software.

### Enzyme assays

The duodenum was maintained on ice and homogenized in 10 volumes of 0.1 M phosphate buffer with 0.002 M EDTA (pH 7.3) using an Ultra Turrax homogenizer (20,000 rpm). Samples were sonicated for 45 s on ice using an Ultrasonic Processor VCX 130. Cellular debris was removed by centrifugation for 15 min at 12,000 G at 4 °C. Then, the supernatant was collected and used for determination of protein concentration by Bradford method.

Citrate synthase and cytochrome C oxidase activity were determined spectrophotometrically according to [49].

### Western blot

Homogenized duodenums, stored at -80 °C with proteases and phosphatase inhibitors, were left on ice for 1 h. 50 µg of the sample were loaded on acrylamide gel posterior to Lowry quantification (550 nm; Tecan M200) and then transferred to a nitrocellulose film. The film was then treated with BSA 5% in TBS-T 0,1% for an hour, washed in PBS and incubated for 12 h with anti-phospho p70 S6K1 (Thr389) (1:1000, Cell signaling) or anti-p70 S6K1 (1:1000, Cell signaling) and anti-β actin (1: 5000, Cell Signaling). For protein detection, we used Rabbit anti-mouse HRP (1:5000; Cell signaling) to identify p70 S6K1 and p70 S6K1 phosphorylated and Mouse anti-β actin (1:5000, Cell Signaling) with luminol. X-ray films were obtained after 1 h exposition in a dark room for S6K1 and S6K1-P, while β actin detection lasted 10 s of exposition. The quantification shown in Fig. 5B corresponds to the sum of all results, obtained after the representative experiment shown in Fig. 5A.

### Gene expression

Total RNA was obtained from the first third of the duodenum of C20D and H20D groups by phenol–chloroform extraction using Trizol. cDNA was then synthesized using 1 μg of RNA and a M-MLV reverse transcription kit (Invitrogen). Relative expression of several genes related to the mTOR pathway was assessed by qPCR (Agilent Technologies Mx3000P RT-PCR System) using the designed primers indicated in Table 3. Data was analyzed by calculating the expression fold change via 2^−ΔΔCt^, and gene expression was normalized to that of two housekeeping genes (*GAPDH* and *B2M*).

**Table 3:** Primers sequence for selected genes used in this study

Gene	5´to 3´	
	Forward	Reverse
*Foxo1*	AGCGTGCCCTACTTCAAGGAT	TTGTCCATGGACGCAGCTCTT
*Mtor*	TTTCCTGCGCAAGATGCTCA	AGCCTTCAGGATAGGCTCCAT
*Eef2*	AAGGCCGCTTCTATGCCTTT	TCCACATGGCACGTCCTCAA
*Eif4e*	GCGCTTGCTTCTAGATTCCGA	AGTGCTCTGGGTTAGCAACCT
*Skar*	AAAGGCCATGGTGCCACTTCA	CCGTGGCTGCAAACTTCATCT
*S6*	CTCTTTTTCGTGACGCCTCCCA	CACCAAGAGCATCAGCGGCTA
*Nrf2*	CTACAGTCCCAGCAGGACATGG	GTTCCTTCTGGAGTTGCTCTTGT

### Statistical analysis

Two independent observers scored the number of cells labeled as positive per villus of each crypt. In order to investigate whether HBOT affects the proliferation dynamics of ISCs, the number of cells positive for BrdU was quantified in 36 randomly selected crypts from the first third of the intestine of each individual (n = 3 per treatment). Permutation test was performed in R software, using a permutation test of 1000 permutation for each analysis. Data from mTOR inhibition by rapamycin and western blot were also analyzed by permutation test. Data distribution was analyzed using a Kolmogorov–Smirnov test. As for the first experiment (HBOT for either 10 or 20 days) we considered a Bonferroni correction, having an α = 0.0125.

## Supplementary Information

**Additional file 1: Figure S1. **HBOT effects on proliferation of ISCs. **A** Total cell number comparison between control small intestines and HBOT small intestines (p = 0.049). **B** Total BrdU + cells within the crypt between control and HBOT small intestines (p = 0.048). Permutation test (10000 permutations). n = 3.

**Additional file 2: Figure S2.** HBOT does not increase S6K1 expression. A Total or phosphorylated S6K1 expression Western-Blot analysis under experimental conditions as indicated. B Pixel intensity analysis of Western-Blot in A, normalized through total S6K1. Asterisks indicate significance (< 0.05). Permutation test. n = 3**.**

## Data Availability

All data is available as figures or supplementary information.

## Abbreviations

HBOT: Hyperbaric oxygen treatment
mTORC1: MTOR complex 1
ISCs: Intestinal stem cells
CR: Caloric restriction
BrdU: Bromodeoxyuridine
COX: Cytochrome c oxidase
CS: Citrate synthase
IF: Immunofluorescence
IHQ: Immunohistochemistry
