# Supplementary figures and images for: Hyperbaric oxygen treatment increases intestinal stem cell proliferation through the mTORC1/S6K1 signaling pathway in Mus musculus
Source: Biol Res. 2023 Jul 13;56:41. doi: 10.1186/s40659-023-00444-3 (PMC10339527; doi:10.1186/s40659-023-00444-3)

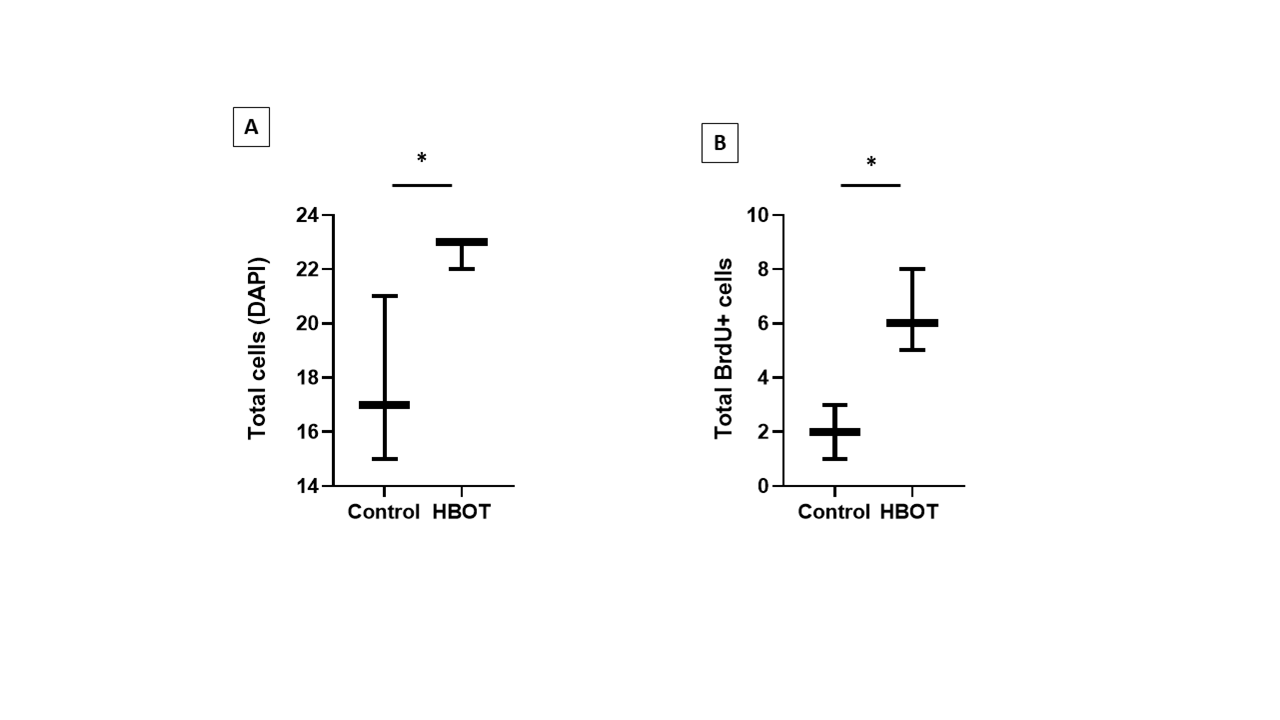

Supplement: Supplementary file 1 — Additional file 1: Figure S1. HBOT effects on proliferation of ISCs. A Total cell number comparison between control small intestines and HBOT small intestines (p = 0.049). B Total BrdU + cells within the crypt between control and HBOT small intestines (p = 0.048). Permutation test (10000 permutations). n = 3. [file 40659_2023_444_MOESM1_ESM.tif]

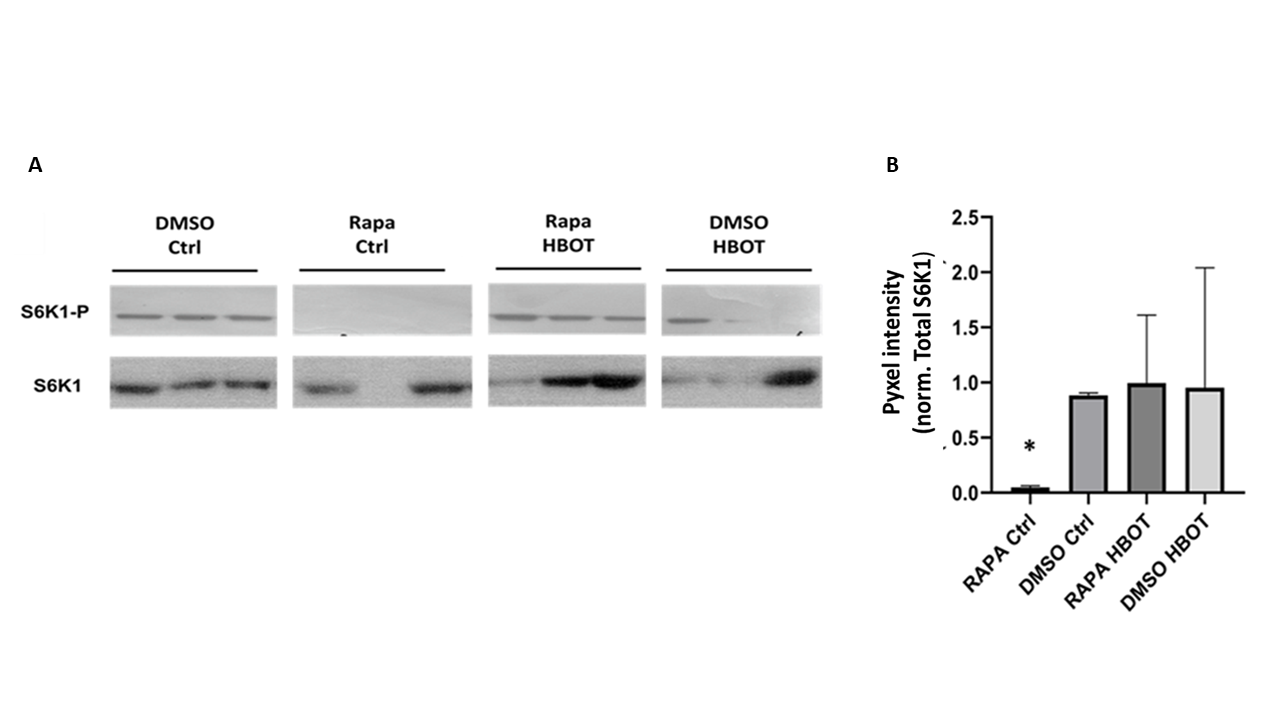

Supplement: Supplementary file 2 — Additional file 2: Figure S2. HBOT does not increase S6K1 expression. A Total or phosphorylated S6K1 expression Western-Blot analysis under experimental conditions as indicated. B Pixel intensity analysis of Western-Blot in A, normalized through total S6K1. Asterisks indicate significance (< 0.05). Permutation test. n = 3. [file 40659_2023_444_MOESM2_ESM.tif]
